# Risk Factors Associated to Mortality in the Indigenous Population with COVID-19 in Mexico

**DOI:** 10.1007/s40615-025-02525-3

**Published:** 2025-06-27

**Authors:** Laura Flores-Cisneros, Rosaura Gutiérrez-Vargas, Carlos Escondrillas-Maya, Brenda Téllez-Flores, Gabriel García-Rodríguez, Ruy López-Ridaura, Christian Zaragoza-Jiménez, Dulce González-Islas

**Affiliations:** 1 Dirección de Información Epidemiológica, Dirección General de Epidemiología, Secretaría de Salud, Ciudad de Mexico, Mexico; 2 Departamento de Vigilancia de Enfermedades de Transmisión Directa, Instituto Mexicano del Seguro Social – Bienestar, Ciudad de Mexico, Mexico; 3 Subsecretaría de Prevención y Promoción de La Salud, Secretaría de Salud, Ciudad de Mexico, Mexico; 4 Dirección General de Información en Salud, Secretaría de Salud, Ciudad de Mexico, Mexico; 5 Heart Failure and Respiratory Distress Clinic, Instituto Nacional de Enfermedades Respiratorias “Ismael Cosío Villegas”, Cardiology ServiceCalzada de Tlalpan 4502 Col Sec XVI, Del Tlalpan, C.P. 14080 Mexico City, Mexico

**Keywords:** COVID-19, Vulnerable populations, Risk factors, Mortality

## Abstract

**Objective:**

During the COVID-19 pandemic, the Indigenous population in Mexico was subject to several factors that caused them to have a higher mortality risk than the non-Indigenous population. These risk factors included language barriers, poverty, comorbidities, inadequate access to health services, lack of social security, limited education, and substandard housing, *inter alia*. This study aims to determine the factors associated with COVID-19 mortality in the Indigenous population in Mexico.

**Study Design:**

Cohort study.

**Methods:**

This study utilized data from the National Epidemiological Surveillance System for Viral Respiratory Disease (SISVER) collected during 2020 and 2021 to examine the population recognized as Indigenous.

**Results:**

A total of 506,956 COVID-19 cases were reported, with 0.75% identified as Indigenous. The logistic regression assessed the combined effect of Indigenous status and each risk factor. Adjusted odds ratios (aORs) with or without interaction terms were reported. The identified risk factors were the following: age of > 60 years × Indigenous (aOR 9.19, CI 95% 6.38–13.2, *p* < 0.001); male × Indigenous (aOR 3.38, CI 95% 2.72–5.53, *p* < 0.001); and time from symptom onset to seeking care > 7 days × Indigenous (aOR 5.86, CI 95% 4.10–8.37, *p* < 0.001).

**Conclusions:**

Belonging to the Indigenous population is a significant risk factor for COVID-19 mortality in Mexico. Although both Indigenous and non-Indigenous groups share common risk factors, the combined effect of Indigenous status and each risk factor reveals greater vulnerability among the former. Significant interactions were observed with age over 60 years, sex, and delays in seeking medical care after the onset of symptoms.

## Introduction

The SARS-CoV-2 pandemic exposed disparities in the diagnosis and progression of COVID-19 among ethnic minorities. Studies in the UK indicated that during the initial phase of the pandemic, a significant proportion of critically ill COVID-19 patients belonged to ethnic groups. Similarly, data from the USA revealed a disproportionate percentage of COVID-19-related infections, hospitalizations, and deaths among African American and Hispanic populations relative to the general population [1].

According to the 2020 population and housing census, approximately 23.2 million people in Mexico aged 3 years and above self-identify as Indigenous, representing 19.4% of the country’s total population in this age range [2]. A Mexican study utilizing data collected from January 2020 to March 2022 found that the COVID-19 mortality rate among Indigenous groups was notably higher than that of non-Indigenous populations. Furthermore, significant delays were observed among Indigenous people in seeking care for COVID-19, as well as a lower probability of survival compared with their non-Indigenous counterparts [3].

Factors such as advanced age, comorbidities, and Indigenous identity have been associated with higher mortality rates or fatal outcomes [4]. In other countries, the mortality rate among Indigenous populations is three times higher than that of the general population [5].

The Indigenous population is particularly vulnerable to health emergencies due to genetic and cultural factors. Moreover, factors such as extreme poverty, residence in marginalized areas, limited access to health services, discrimination, and organizational barriers to utilizing services further disadvantage this group [6–8].

This study aimed to determine the factors associated with COVID-19 mortality in the Indigenous population in Mexico during 2020 and 2021, as reported by SISVER (National Epidemiological Surveillance System for Viral Respiratory Disease).

## Materials and Methods

### Study Design and Setting

A retrospective cohort study was conducted for this research. COVID-19 information regarding Mexico was obtained through the SISVER, which includes viral respiratory disease sentinel surveillance units (USMER) and non-USMER units. In the present study, data were collected from the SISVER online platform.

### Data Collection

Information was collected after each state uploaded data from first-, second-, and third-level medical care units to the SISVER online platform.

This study included suspected cases of viral respiratory disease based on an operational definition: individuals of any age who, within the past 7 days, experienced cough, fever, or headache, with at least two of these symptoms, accompanied by at least one of the following signs or symptoms: dyspnea, arthralgia, myalgia, odynophagia, rhinorrhea, conjunctivitis, or chest pain. This definition was approved by the National Epidemiological Surveillance Committee [9].

The study included all cases aged > 18 years reported in the database from March 1, 2020, to December 31, 2021. A total of 6,506,956 cases were included in SISVER. Positive COVID-19 cases were confirmed through the presence of SARS-CoV-2 using real-time reverse-transcription polymerase chain reaction method, antigen positivity, and clinical epidemiological assessment or epidemiological association.

A patient was identified as Indigenous if they self-identified as Indigenous and spoke any Indigenous language, irrespective of whether they also spoke Spanish.

The primary objective of this study was to determine the risk factors associated with mortality in the Indigenous population.

### Statistical Analysis

In the descriptive analysis, the categorical variables were expressed as frequencies and percentages. The Shapiro–Wilk test was used to assess the normality of continuous variables; normal continuous variables were expressed as mean and standard deviation, while non-normal variables were expressed as medians and percentiles (25–75). Comparisons among study groups were conducted using the *X*^2^ test or Fisher’s exact test for categorical variables and the unpaired Student’s *t*-test or Mann–Whitney *U* test for continuous variables.

A logistic regression model was performed to evaluate the association between risk factors and mortality among Indigenous and non-Indigenous populations. Interaction terms with Indigenous status (e.g., sex × Indigenous, age × Indigenous, comorbidities × Indigenous) were included to assess whether the effects of these variables differed by Indigenous status. In the multivariate model, the combined effect of Indigenous status and each risk factor was estimated. Variables with a *p*-value < 0.05 in the bivariate analysis were included in the multivariate model, along with their interaction terms. Odds ratios (OR) and 95% confidence intervals (CI) were estimated. All *p*-values presented were two-sided; a *p*-value < 0.05 was considered statistically significant. Statistical analyses were performed using Stata software, version 16.0 (StataCorp, College Station, Texas, USA).

## Results

A total of 6,506,956 COVID-19 cases were reported, of which 49,023 were identified as Indigenous (0.75%). The median ages of the overall population, Indigenous population, and non-Indigenous population were 38 (28–51), 42 (30–57), and 38 (28–51), respectively. In both Indigenous and non-indigenous groups, more than half of the population was under 60 years of age. The predominant gender in both groups was female, with a prevalence of 50.7% in the Indigenous group and 53.0% in the non-Indigenous group (*p* < 0.001).

The Indigenous population received a higher proportion of medical attention from the Ministry of Health than the non-indigenous population (68.8% vs. 40.9%, *p* < 0.001). Further, the central and southern geographic regions exhibited the highest concentrations of Indigenous population (central: 47.7% vs. 44.2%; south: 24.1% vs. 11.1%, *p* < 0.001) (Table 1).

**Table 1:** Sociodemographic and clinical characteristics in indigenous or non-indigenous patients

	**Total**	**Indigenous population**	**Not-indigenous population**	***p*-value**
	***n* = 6,506,956**	***n* = 49,023**	***n* = 6,457,933**	
**Age**	38 (28–51)	42 (30–57)	38 (28–51)	< 0.001
< 60 years	5,656,008 (86.9)	38,794 (79.1)	5,617,214 (87.0)	< 0.001
≥ 60 years	850,948 (13.1)	10,229 (20.8)	840,719 (13.0)	
**Sex**				
Female	3,450,606 (53.0)	24,878 (50.7)	3,425,728 (53.0)	< 0.001
Male	3,056,350 (47.0)	24,145 (49.2)	3,032,205 (46.9)	
Migrant	3867 (22.2)	22 (31.9)	3845 (22.2)	0.053
**Ocupation**				
Health personal	495,055 (7.6)	3,871 (7.9)	491,184 (7.6)	< 0.001
Household	1,761,977 (27.1)	17,456 (35.6)	1,744,521 (27.0)	
Driver	78,754 (1.2)	693 (1.4)	78,061 (1.2)	
Merchant	140,257 (2.2)	1600 (3.3)	138,657 (2.1)	
Employee	2,743,194 (42.2)	11,467 (23.4)	2,731,727 (42.3)	
Other	1,286,143 (19.9)	13,929 (28.42)	1,273,564 (19.72)	
**Health sector**				
Ministry of Health and Assistence	2,673,892 (41.1)	33,727 (68.8)	2,640,165 (40.9)	< 0.001
Mexican rance Institute	3,202,139 (49.2)	11,902 (24.3)	3,190,237 (49.4)	
ISSSTE	183,372 (2.8)	1656 (3.4)	181,716 (2.8)	
Private	289,991 (4.5)	784 (1.6)	289,207 (4.5)	
State	68,601 (1.0)	193 (0.4)	68,408 (1.1)	
Mexican Petroleum Company	38,807 (0.6)	112 (0.2)	38,695 (0.6)	
Other	50,154 (0.8)	649 (1.3)	49,505 (0.8)	
**Geographic region**				
Center	2,881,267 (44.2)	23,273 (47.7)	2,857,994 (44.2)	< 0.001
Center-West	1,365,572 (20.9)	9,113 (18.6)	1,356,459 (21.0)	
North	1,531,225 (23.5)	4798 (9.8)	1,526,427 (23.6)	
South	728,892 (11.2)	11,839 (24.1)	717,053 (11.1)	
**Comorbidities**				
Diabetes	582,155 (8.9)	6927 (14.1)	575,228 (8.9)	< 0.001
Hypertension	785,125 (12.1)	7644 (15.6)	777,481 (12.0)	< 0.001
Obesity	645,848 (9.9)	6682 (13.6)	639,166 (9.9)	0.008
Asthma	122,980 (1.9)	970 (2.0)	122,010 (1.9)	0.008
COPD	45,032 (0.7)	906 (1.8)	44,126 (0.7)	< 0.001
Immunosuppression	36,827 (0.6)	412 (0.8)	36,415 (0.6)	< 0.001
HIV/AIDS	18,302 (0.3)	156 (0.3)	18,146 (0.3)	0.003
Chronic kidney disease	63,503 (1.0)	694 (1.4)	62,809 (1.0)	< 0.001
Heart disease	65,3371 (1.0)	677 (1.4)	64,660 (1.0)	< 0.001
Smoking	363,900 (5.6)	2147 (4.4)	361,753 (5.6)	< 0.001
**Clinical characteristics**				
Pregnant	56,964 (1.1)	1296 (4.2)	55,668 (1.1)	< 0.001
Dyspnea	1,085,832 (16.6)	13,533 (27.6)	1,072,299 (16.6)	< 0.001
Intubated	81,432 (12.1)	1186 (11.8)	80,246 (12.1)	0.441
Admitted to ICU	51,100 (7.6)	969 (9.6)	50,131 (7.5)	< 0.001
**Time from symptom onset to seeking care**	3 (1–4)	3 (2–5)	3 (1–4)	< 0.001
**Time from symptom onset to seeking care ≥ 7 days**	729,064 (11.2)	7695 (15.7)	721,369 (11.2)	< 0.001
**Non-survival**	318,517 (4.9)	5,012 (10.2)	313,505 (4.8)	< 0.001
**COVID-19 vaccinated**	1,503,111 (23.1)	11,014 (22.5)	1,492,097 (23.2)	0.001

*COPD* chronic obstructive pulmonary disease, *ICU* intensive care unit, *ISSSTE* Institute for Social Security and Services for State Employees

Data are presented as *n* (%) or median (p25–p75), chi-square test, and Man-Whitney *U* test

The Indigenous group exhibited a higher prevalence of comorbidities than the non-Indigenous group. Specifically, the Indigenous group had higher rates of diabetes (14.1% vs. 8.9%, *p* < 0.001), hypertension (15.6% vs. 12.0%, *p* < 0.001), and obesity (13.6% vs. 9.9%, *p* < 0.001). Additionally, the Indigenous group had a higher rate of admission to the intensive care unit (9.6% vs. 7.5%, p < 0.001) and exhibited a longer time between symptom onset and seeking care (≥ 7 days) compared with the non-indigenous group (15.7% vs. 11.2%, *p* < 0.001). Further, the proportion of non-survivors was higher in the Indigenous group than in the non-Indigenous population (10.2% vs. 4.8%, *p* < 0.001) (Table 1).

Table 2 presents the risk factors for mortality based on univariate logistic regression according to population, demonstrating that age and healthcare institutions were associated with mortality. Table 3 shows the univariate model with each variable’s Indigenous population interaction term. The Indigenous population was at a higher risk of death than the non-Indigenous population (OR 2.36, CI 95% 1.65–3.36, *p* < 0.001). The interaction variables included age, sex, IMSS, ISSSTE, geographic region (central and north), vaccination status, and dyspnea.

**Table 2:** Risk factors associated to mortality according to population study (univariated model)

	**Indigenous population**			**Not-indigenous population**		
**Variable**	**OR**	**95% CI**	***p*-value**	**OR**	**95% CI**	***p*-value**
**Age, years**						
≥ 60	9.90	9.30–10.55	< 0.001	14.14	14.04–14.25	< 0.001
< 60	Reference					
**Sex**						
Male	1.72	1.62–1.82	< 0.001	1.86	1.84–1.87	< 0.001
Female	Reference					
**Ocupation**						
Household	15.4	1.2–21.1	< 0.001	11.6	11.3–12.0	< 0.001
Driver	17.9	12.3–26.1	< 0.001	10.4	10.0–10.8	< 0.001
Merchant	20.3	14.4–28.5	< 0.001	10.0	9.6 −10.3	< 0.001
Employee	15.7	11.4–21.6	< 0.001	5.8	5.6–6.0	< 0.001
Other	2.7	2.0–3.8	< 0.001	1.8	1.8–1.9	< 0.001
Health personal	Reference					
**Health sector**						
Mexican Social Insurance Institute	1.11	1.04–1.19	< 0.001	1.69	1.68–1.71	< 0.001
ISSSTE	2.23	1.96–2.53	< 0.001	3.98	3.92–4.04	< 0.001
Private	0.69	0.52–0.91	< 0.001	0.65	0.63–0.66	< 0.001
State	0.98	0.60–1.60	< 0.001	1.93	1.87–1.99	< 0.001
Mexican Petroleum Company	1.59	0.93–2.71	< 0.001	3.25	3.99–4.21	< 0.001
Other	3.76	3.15–4.47	< 0.001	4.10	4.0–4.2	< 0.001
Ministry of Health and Assistance	Reference					
**Geographic region**						
Center-West	0.61	0.55–0.67	< 0.001	1.10	1.09–1.11	< 0.001
North	0.84	0.75–0.94	< 0.001	1.11	1.10–1.12	< 0.001
South	1.29	1.20–1.38	< 0.001	1.12	1.10–1.13	< 0.001
Center	Reference					
**Comorbidities**						
Diabetes	4.51	4.22–4.81	< 0.001	7.20	7.214–7.26	< 0.001
COPD	5.15	4.48–5.92	< 0.001	8.99	8.81–9.18	< 0.001
Immunosuppression	2.85	2.27–3.57	< 0.001	4.88	4.76–5.01	< 0.001
Hypertension	4.26	4.00–3.6	< 0.001	6.86	6.81–6.92	< 0.001
HIV/AIDS	1.29	0.80–2.06	< 0.001	1.73	1.64–1.83	< 0.001
Heart disease	4.19	3.55–4.94	< 0.001	6.52	6.40–6.64	< 0.001
Obesity	1.98	1.84–2.13	< 0.001	2.52	2.50–2.54	< 0.001
Chronic renal failure	5.59	4.78–6.54	< 0.001	11.84	11.64–12.04	< 0.001
**COVID-19 vaccinated**	0.29	0.26–0.33	< 0.001	0.18	0.18–0.18	< 0.001
**Time from symptom onset to seeking care ≥ 7 days**	3.50	3.28–3.73	< 0.001	4.88	4.84–4.92	< 0.001
**Dyspnea**	57.12	50.93–64.06	< 0.001	75.66	74.66–76.64	< 0.001

*COPD* chronic obstructive pulmonary disease, *ICU* intensive care unit, *ISSSTE* Institute for Social Security and Services for State Employees

**Table 3:** Logistic regression with indigenous population interaction term

Variable	OR	95% CI	*p*-value	Interaction coefficient	95% CI	*p*-value
**Indigenous**	2.36	1.65–3.36	< 0.001	NA	NA	NA
**Age, years**						
≥ 60	4.33	4.29–4.38	< 0.001	0.89	0.82–0.97	0.010
< 60	Reference					
**Sex**						
Male	1.87	1.85–1.89	< 0.001	0.87	0.79–0.93	0.007
Female	Reference					
**Ocupation**						
Household	3.77	11.18–21.15	< 0.001	1.02	0.73 − 1.44	0.872
Driver	3.59	3.44–3.75	< 0.001	1.27	0.83–1.94	0.254
Merchant	4.52	4.35–4.70	< 0.001	1.00	0.69 − 1.46	0.983
Employee	1.70	1.65–1.76	< 0.001	1.05	0.73–1.50	0.787
Other	2.97	2.87–3.07	< 0.001	1.14	0.81–1.61	0.440
Health personal	Reference					
**Health sector**						
Mexican Social Insurance Institute	1.99	1.97–2.01	< 0.001	0.52	0.47–0.57	< 0.001
ISSSTE	1.83	1.80–1.87	< 0.001	0.71	0.60–0.85	< 0.001
Private	0.67	0.65–0.69	< 0.001	1.08	0.77–1.52	0.646
State	1.59	1.53–1.66	< 0.001	0.72	0.38–1.35	0.315
Mexican Petroleum Company	1.22	1.17–1.28	< 0.001	0.57	0.28–1.14	0.117
Other	1.58	1.52–1.63	< 0.001	0.86	0.69–1.08	0.218
Ministry of Health and Assistance	Reference					
**Geographic region**						
Center-West	0.85	0.84–0.86	< 0.001	0.79	0.70–0.88	< 0.001
North	1.04	1.03–1.05	< 0.001	0.82	0.71–0.94	0.006
South	1.13	1.12–1.5	< 0.001	0.97	0.88–1.06	0.525
Center	Reference					
**Comorbidities**						
Diabetes	1.39	1.37–1.40	< 0.001	0.97	0.88–1.08	0.678
COPD	1.01	0.98–1.04	0.331	1.08	0.90–1.29	0.363
Immunosuppression	1.36	1.31–1.41	< 0.001	0.94	0.70–1.28	0.730
Hypertension	1.17	1.16–1.19	< 0.001	0.99	0.89–1.09	0.879
HIV/AIDS	0.97	0.91–1.04	0.513	0.86	0.48–1.53	0.614
Heart disease	1.19	1.17–1.22	< 0.001	1.07	0.88–1.31	0.461
Obesity	1.18	1.16–1.20	< 0.001	1.07	0.96–1.19	0.217
Chronic renal failure	2.02	1.98–2.07	< 0.001	0.28	0.28–0.29	0.771
Comorbidities ≥ 1	1.56	1.54–1.59	< 0.001	0.91	0.80–1.03	0.165
Without comorbidities	Reference					
**Vaccinated**	0.28	0.28–0.29	< 0.001	1.47	1.29–1.67	< 0.001
**Time from symptom onset to seeking care ≥ 7 days**	1.57	1.56–1.59	< 0.001	0.92	0.85–1.00	0.060
**Dyspnea**	34.6	34.1–35.1	< 0.001	0.80	0.71–0.90	< 0.001

*COPD* chronic obstructive pulmonary disease, *ICU* intensive care unit, *ISSSTE* Institute for Social Security and Services for State Employees

**Fig. 1:**
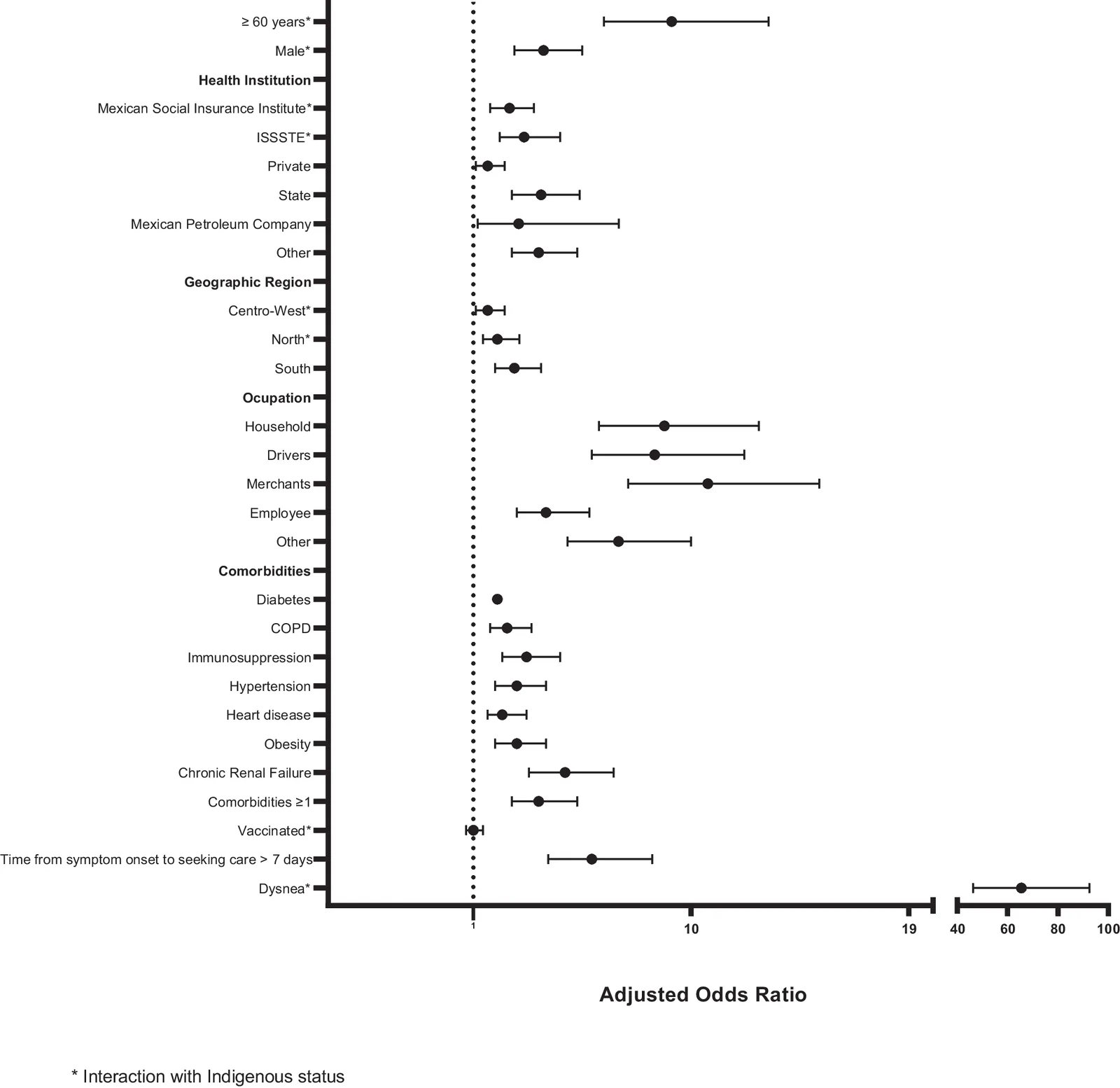
Multivariate model of the combined effect of being Indigenous and each risk factor

Figure 1 illustrates the multivariate model assessing the combined effect of Indigenous status and each risk factor.

Adjusted odds ratios (aORs) with or without interaction terms were reported. The risk factors associated with mortality were as follows: age of > 60 years × Indigenous (aOR 9.19, CI 95% 6.38–13.2, *p* < 0.001) and male × Indigenous (aOR 3.88, CI 95% 2.72–5.53, *p* < 0.001). Regarding health institutions, IMSS × Indigenous (aOR 2.47, CI 95% 1.72–3.55, *p* < 0.001), ISSSTE × Indigenous (aOR 3.11, CI 95; 2.11–4.58, *p* < 0.001), private (aOR 1.59, CI 95% 1.11–2.27, *p* = 0.010), state (aOR 3.77, CI 95% 2.63–5.39, *p* < 0.001), and PEMEX (aOR 2.90, CI 95% 2.02–4.15, *p* < 0.001). Concerning geographic regions, central-west × Indigenous (OR 1.59, CI 95% 1.10–2.30, *p* = 0.012), north × Indigenous (aOR 2.03, CI 95% 1.39–2.95, *p* < 0.001), and south (aOR 2.68, CI 95% 1.88–3.83, *p* < 0.001). Regarding occupation, household (aOR 8.91, CI 95% 6.22–12.76, *p* < 0.001), drivers (aOR 8.48, CI 95% 5.91–12.17, *p* < 0.001), and merchants (aOR 10.68, CI 95% 7.45–15.31, *p* < 0.001). In terms of comorbidities, diabetes (aOR 2.03, CI 95% 2.01–2.05, *p* < 0.001), chronic obstructive pulmonary disease (COPD) (aOR 2.39, CI 95% 1.67–3.41, *p* < 0.001), immunosuppression (aOR 3.21, CI 95% 2.24–4.59, *p* < 0.001), hypertension (aOR 2.77, CI 95% 1.94–3.96, *p* < 0.001), heart disease (aOR 2.23, CI 95% 1.56–3.19, *p* < 0.001), obesity (aOR 2.79, CI 95% 1.95–3.99, *p* < 0.001), chronic renal failure (aOR 4.78, CI 95% 3.35–6.82, *p* < 0.001), and the presence of more than one comorbidity (aOR 3.70, CI 95% 2.59–5.28, *p* < 0.001). Additionally, time from symptom onset to seeking care > 7 days × Indigenous (aOR 5.86, CI 95% 4.10–8.37, *p* < 0.001) and dyspnea (aOR 65.46, CI 95% 46.3–92.4, *p* < 0.001).

## Discussion

While numerous studies have focused on the effects of the COVID-19 pandemic on racial minorities or ethnic groups, evidence reflecting the vulnerability and risk of the Indigenous population during health emergencies is still limited; hence, this study explored the main risk factors for mortality in the Indigenous population in Mexico.

Our findings indicate that age is a risk factor for both the Indigenous and non-Indigenous populations. However, we observed that Indigenous individuals over 60 years of age had a risk of death nine times higher than that of non-Indigenous individuals under 60 years of age. This aligns with findings from other studies, which indicate that while all age groups are at risk of contracting SARS-CoV-2, the mortality risk is particularly pronounced among those over 60 [10]. Soto-Cabezas et al. found that mortality due to COVID-19 among Amazonian Indigenous people was similar among those under 59 years of age, but it was significantly higher among older adults [11].

Other studies on Indigenous populations in Mexico have revealed that older adults are more likely to die from COVID-19, especially those who received outpatient medical care but were not hospitalized [12]. Similarly, Serván-Mori et al. associated age with a higher probability of hospitalization and death, observing a substantial increase in effect size with advancing age groups [8].

Concerning gender, Ibarra-Nava et al. and Soto-Cabezas et al. concluded that the male gender and the presence of comorbidities are significant predictors of death among COVID-19 patients [11, 12] Our study demonstrated an interaction between Indigenous status and sex; Indigenous males have a risk of death 3.88 times higher than non-Indigenous females. Regarding comorbidities, we analyzed the combined effect of Indigenous status and the presence of comorbidities. Being Indigenous and having conditions such as diabetes, COPD, immunosuppression, heart disease, obesity, chronic renal failure, or multiple comorbidities were found to significantly increase the risk of death compared to non-Indigenous individuals without comorbidities.

The analysis in this study suggests that the Indigenous population residing in the southern region of Mexico is at a higher risk of COVID-19 mortality. Previous studies in Mexico have identified geographic areas with significant public health challenges, including difficulties in accessing health services, particularly in regions where Indigenous communities are concentrated. Health issues prevalent in this population include high rates of premature births, a high incidence of infectious diseases (such as respiratory infections, tuberculosis, and zoonotic diseases), malnutrition, and chronic diseases such as diabetes mellitus, obesity, hypertension, and heart disease [13]. Data from the National Council for the Evaluation of Social Development Policy (CONEVAL) indicated in 2020 that municipalities with 80% or more of adults living in poverty were located in specific areas of the south and Indigenous regions [14]. Similar results were found by Rodrigues-Novaes in the Brazilian Indigenous population with severe acute respiratory syndrome, where deaths were concentrated in geographic regions such as the states of Amazonas and Mato Grosso do Sul, located in the North and Midwest of Brazil. Furthermore, Indigenous individuals residing in rural areas and having lower levels of education faced a higher risk of mortality than those living in urban areas and having higher levels of education [15].

Another study concluded that individuals living in municipalities with higher poverty rates had a greater risk of mortality from COVID-19 than those residing in municipalities with lower poverty rates [16], which may explain the association of mortality with the Indigenous population. Our study’s results indicated that the Indigenous individuals most at risk of mortality were primarily tradespeople, stay-at-home workers (including housewives, older adults, and even young children who stayed at home, and drivers). Jobs that cannot be performed at home and require high levels of contact with others were associated with a higher risk of infection. These findings are partially supported by a cohort study of workers in England and Wales on occupation and vaccine uptake, which indicated that jobs with the highest vulnerability to severe COVID-19 were in commerce, processing, manufacturing, sales, and customer services [17]. Another study found that older individuals faced a higher mortality risk when living with delivery or postal workers or with individuals in occupations outside the home, and cab and bus drivers faced a relative risk of COVID-19 mortality four times higher than that faced by workers in other occupations when adjusted for basic demographic characteristics [18].

Mexico has a high prevalence of chronic diseases, which represents an important risk factor for COVID-19 mortality, alongside Indigenous identity [19]. Our study’s results indicated that chronic renal failure, immunosuppression, obesity, hypertension, COPD, heart disease, and diabetes posed significant mortality risks in the Indigenous population compared with the non-Indigenous population. This is in line with Serván-Mori et al., who found that medical comorbidities, including diabetes, obesity, hypertension, and chronic kidney disease, are associated with higher odds of hospitalization, death, and early mortality. COPD, early mortality, and cardiovascular disease were linked to increased odds of hospitalization [8].

This study’s data revealed a significant association between the presence of multiple comorbidities and mortality, a finding supported by Soto et al. in their study on Peruvian Amazonian Indians, which showed that individuals with two risk conditions had a mortality risk 1.70 times higher than those with one condition and 4.51 times higher than those with no risk conditions [11].

Multiple pieces of evidence indicate that racial and ethnic minorities face significant barriers to accessing quality health care and have less access to primary care providers. Additionally, high hospitalization costs related to COVID-19 disproportionately impacted low-income ethnic minorities. Therefore, access to and quality of care are critical factors influencing the impact of COVID-19 on the Indigenous population [12, 20]. Our study’s findings suggest a greater likelihood of death among Indigenous individuals who sought care later (more than 7 days after symptom onset) than among those who sought timely care. This is consistent with previous studies in Mexico, where Indigenous individuals exhibited longer delays in seeking care for COVID-19 infection and demonstrated a lower probability of survival than non-Indigenous populations [3]. Similarly, Lee et al. concluded that Indigenous individuals and people of color, as well as those with disabilities, may experience discrimination in medical care, leading to delays or inferior care, *inter alia* [21].

Vaccination against COVID-19 became crucial in the global fight against the pandemic since the approval of safe and effective vaccines in December 2020. These vaccines have been shown to minimize the risk of symptomatic SARS-CoV-2 infection and significantly reduce disease severity, thereby decreasing COVID-19 morbidity and mortality [17]. Our current study suggests no association between the combined effects of vaccination status and Indigenous identity, indicating that vaccines offer a similar level of protection against mortality for both Indigenous and non-Indigenous populations.

COVID-19 can progress to pneumonia, severe acute respiratory distress syndrome, and death in extreme cases, particularly among vulnerable groups such as African Americans, Hispanics, and Indigenous populations. A study conducted on Brazilian Indigenous individuals during the first 57 weeks of the pandemic evaluated 17 symptoms, finding that only three were associated with an increased risk of death: oxygen saturation below 95%, dyspnea, and respiratory distress. The data, particularly regarding dyspnea, suggest that Indigenous individuals presenting this symptom were more likely to die than those with other analyzed mortality factors [22].

This study has important limitations; the records analyzed were collected from medical units distributed throughout the country, and cases of illness outside these units were not included. Additionally, 100% of severe cases were sampled for RT-PCR or antigen diagnosis, while cases with mild symptoms were only sampled at about 10%. This overrepresentation of severe cases may introduce selection bias, potentially inflating mortality estimates and limiting the generalizability of the results. Due to the underrepresentation of the Indigenous population, as the data is only from medical units in the sample of cases, there may be selection bias that could distort the risk estimates.

Furthermore, the vaccination variable was based on self-reporting, where participants answered “yes” or “no” during the medical interview, without confirmation from an official source. Despite these limitations, this study has several strengths. Research into the effects of COVID-19 on the Indigenous population is scarce; therefore, the findings from this study, drawn from a large sample size, reduce confounding factors, allowing for the identification of characteristics that signify a higher risk of death in this vulnerable group.

## Conclusion

This study highlights that belonging to an Indigenous population represents a significant risk factor for mortality in Mexico. While both Indigenous and non-Indigenous populations share common risk factors for COVID-19 mortality, our multivariate model assessing the combined effect of Indigenous status and each risk factor reveals that the Indigenous population is more vulnerable than their non-Indigenous counterpart. Furthermore, significant interactions were observed between Indigenous status and individuals being over 60 years of age, sex, and the time elapsed between symptom onset and seeking medical care.

In summary, this study’s findings support the conclusion that ethnic minority status plays a crucial role as a social determinant in health outcomes associated with COVID-19, likely due to its connection with other social factors influencing health, independent of variables such as age, gender, occupation, vaccination status, and the interval between the onset of symptoms and the presenting of severe complications, such as dyspnea. Other social factors may include housing conditions, employment, socioeconomic status, general health status, and racism.

The results of this study underscore the importance of conducting additional research to comprehensively understand the social determinants of health. They highlight the need to explore additional variables, such as language barriers, misinformation, and lack of trust in public services, which can contribute to the increased risk of contracting COVID-19 in Indigenous populations.

Moreover, results linked to this population segment in the context of the COVID-19 pandemic must be scrutinized, enabling the development of strategies and specific treatments to mitigate the health disparities affecting these minority groups.

## Data Availability

Data will be made available upon request.

## Abbreviations

aOR: Adjusted odds ratio
COPD: Chronic obstructive pulmonary disease
IMSS: Mexican Social Insurance Institute (Instituto Mexicano del Seguro Social)
ISSSTE: Institute for Social Security and Services for State Employees (Instituto de Seguridad y Servicios Sociales de los Trabajadores del Estado)
OR: Odds ratio
PCR: Real-time reverse-transcription polymerase chain reaction
PEMEX: Mexican Petroleum Company (Petróleos Mexicanos)
SISVER: National Epidemiological Surveillance System for Viral Respiratory Disease (Sistema de Vigilancia Epidemiológica de Enfermedades Respiratorias)
SSA: Ministry of Health (Secretaría de Salud)
USMER: Sentinel Surveillance Units (Unidades de Salud Monitoras de Enfermedad Respiratoria)
